# Supplementary figures and images for: A simplified function-first method for the discovery and optimization of bispecific immune engaging antibodies
Source: PLoS One. 2023 Jun 22;18(6):e0273884. doi: 10.1371/journal.pone.0273884 (PMC10286961; doi:10.1371/journal.pone.0273884)

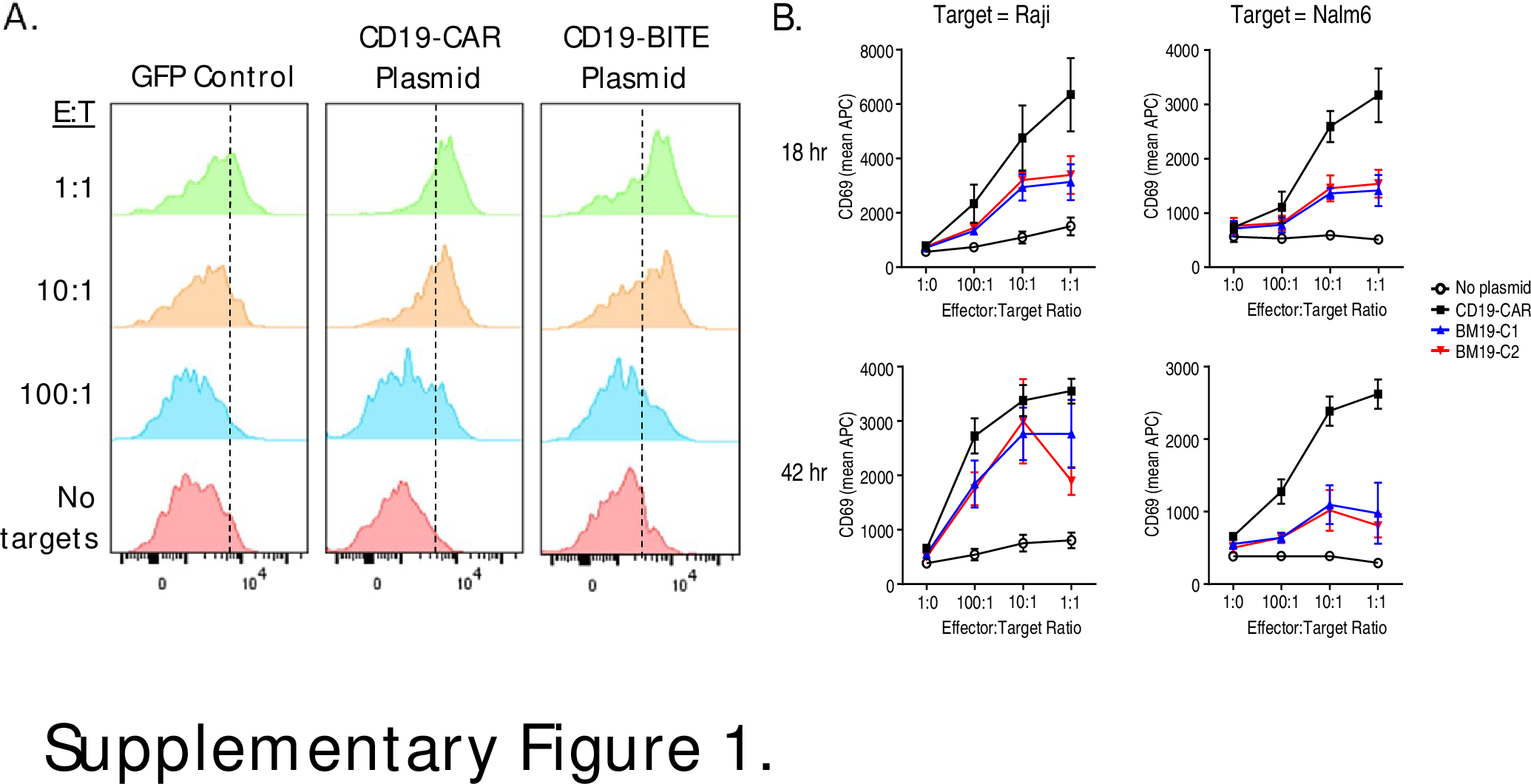

Supplement: S1 Fig — (A) Human Jurkat T cells were electroporated with CD19-targeted BITE or CAR plasmids as described in the methods section. Following recovery, electroporated Jurkat cells were then placed in co-culture with fluorescently labelled CD19-expressing target cells at various effector to target ratios and incubated at 37°C overnight. Co-cultures were stained with anti-human CD69-APC and analyzed via flow cytometry. Results are representative of 3 repeated experiments. (B) The mean fluorescent intensity for CD69-APC staining on gated Jurkat cells is shown for Jurkat-CD19-BITE or Jurkat-CD19-CAR cells in co-culture with CD19+ Raji or NALM6 cells for 18 hours (top) or 42 hours (bottom). Graphs show the mean result from 3 experiments repeated in duplicate +/- standard error of the mean (SEM). (TIF) [file pone.0273884.s001.tif]
