# Supplementary material for: A simplified function-first method for the discovery and optimization of bispecific immune engaging antibodies
Source: PLoS One. 2023 Jun 22;18(6):e0273884. doi: 10.1371/journal.pone.0273884 (PMC10286961; doi:10.1371/journal.pone.0273884)
Supplement: Attachment — Submitted filename: BITE Reviewer response 010323.docx [file pone.0273884.s012.docx]

*Reviewer #1: In the mansucript ‘A Simplified Function-First Method for the Discovery and Optimization of Bispecific Immune Engaging Antibodies’, Shepherd et al. establish a cost effective approach for generating novel BiTE sequences and assessing functional bioactivity bypassing the need for purification. They have generated a bi-modular plasmid and established BiTE-J platform for functional screening. The study is informative, and the findings are relevant for the readership of PLOS ONE.' The study design and experimental approaches are appropriate, and the data generally supports the conclusion. The authors need to address some conceptual and technical concerns in the current version of the manuscript before its publication. The issues are listed below.*

We appreciate Reviewer 1s comments and belief that this manuscript meets the standards for PLOS ONE publication. We have endeavoured to address the remaining concern listed here

*The data in Figure 2, in its present form, should be moved to supplementary*.

We agree that the data shown in what was previously Figure 2, while important, is not the central focus of the text. The figure has been moved to supplementary and the paper has been changed to reflect that.

*The authors state that ‘The addition of non-transfected HEK293T supernatant as a control had no effect on Jurkat activation.’ It would be helpful to include this data.*

Non-transfected HEK293T media is now included as a control in Figure 2B (Previously 3B). This data should be sufficient to show the lack of effect from transfected HEK293T media

*Although the authors have quantified T cell activation and Target Cell Growth, have the authors considered assessing the cytotoxicity of T cells such as by quantifying the Tumor cell lysis and T cell mediated cytotoxicity?*

Given that this paper demonstrates a method of screening potential BiTE candidates, we believe that the Incucyte data shown is robust enough to show that screened BiTEs effectively activate T-cells against target cells and have no off-target effects. When viewing the supplementary videos provided, it is clearly shown that target cell killing begins as early as 24 hours, and is clearly shown to be an effective antigen-specific response. We feel that these videos and the accompanying data demonstrate t cell cytotoxicity without the need for radioactive isotopes or the like. Additionally, as this method removes the need for purification and quantification during screening, we feel that assays measuring tumor cell lysis such a chromium release assay would require the context provided by antibody quantification.

*It is not immediately clear what E:T ratios were used for experiments in Figure 5*.

E:T ratios are included now included in the figure description of Figure 4 (previously Figure 5). This should clarify the data shown.
